# Network-Level Organization of Systemic Inflammation Reflects Early Alzheimer’s-Like Behavioral Changes

**DOI:** 10.21203/rs.3.rs-7482654/v1

**Published:** 2025-10-14

**Authors:** Macy A. Seijo, Anisha Banerjee, Caesar M. Hernandez

**Affiliations:** University of Alabama at Birmingham; University of Alabama at Birmingham; University of Alabama at Birmingham

**Keywords:** systemic inflammation, inflammation networks decision making, motivation, executive function

## Abstract

Systemic immune alterations are increasingly recognized as features of Alzheimer’s disease (AD), yet their network-level organization in preclinical models is poorly understood. We profiled 66 circulating cytokines and growth factors in young adult TgF344-AD and wild-type rats, reduced the data into five inflammatory profiles via principal component analysis, and mapped these profiles onto protein–protein interaction networks. Multivariate analyses revealed genotype- and sex-dependent network organization, with distinct modules enriched for extracellular matrix-linked interleukin signaling or systemic pro-inflammatory cytokine receptor signaling. Regression analyses controlling for genotype and sex linked these networks to specific behavioral domains: extracellular matrix-associated interleukins predicted altered intertemporal choice, whereas pro-inflammatory cytokine receptor signaling correlated with reduced motivation. These findings provide evidence consistent with systemic inflammatory network remodeling at prodromal stages in a preclinical AD model of AD-like pathology and outline a mechanistically interpretable analytical framework with clear translational potential for integrating peripheral immune signatures with behavioral outcomes across species.

## Introduction

Alzheimer’s disease (AD) is a multifactorial neurodegenerative disorder in which neuroinflammation is increasingly recognized as a central driver of pathology. Although classically studied within the brain, systemic immune perturbations also emerge early in disease and may influence or reflect central pathology. In humans, peripheral cytokine and growth factor changes have been associated with cognitive decline, neuropsychiatric symptoms, and AD risk^1–3^, yet most studies focus on individual molecules. Such approaches overlook the fact that the immune system operates as a coordinated, dynamic network. Network-level analyses, capturing the relationships and topologies among multiple immune mediators, offer a richer, systems-level view that can identify functional modules relevant to disease. Here, we apply this framework to the TgF344-AD rat model at prodromal stages, integrating serum measures, principal component analysis, and protein-protein interaction mapping with behavioral phenotyping to define translatable systemic immune network signatures of early AD-related dysfunction.

Evidence from both human and preclinical studies indicates that systemic immune alterations are detectable well before overt cognitive decline. Circulating inflammatory proteins are elevated in transgenic models and in patients at late^4^ and early stages^2,3^ of disease, suggesting that peripheral immune dysregulation may precede or parallel central pathology. Large-scale proteomic studies in humans have applied network-based approaches to plasma, revealing coordinated protein modules associated with extracellular matrix biology, proteostasis, and immune signaling^5^. Similarly, in mouse models of AD, protein-protein interaction (PPI) analyses have been used to map brain proteome alterations and their functional network organization^6–8^. However, no studies to date have applied a network framework to serum inflammatory proteins in preclinical AD models, leaving the systemic dimension of disease-related immune network organization largely unexplored. By focusing on targeted cytokine and growth factor panels, the present study begins to address this gap, enabling mechanistic interpretation of coordinated peripheral immune changes and their relationships to early behavioral dysfunction.

Although the primary neuropathological features of AD reside in the brain, early disease is also characterized by subtle but measurable behavioral changes, including alterations in decision making, motivation, and affective regulation^9–13^. These domains can be assessed in rodent models using operant tasks that are sensitive to early cognitive and emotional dysfunction^14,15^. The TgF344-AD (TgAD) rat model^16^ expresses human-like amyloid and tau pathology and displays both neuroinflammation and behavioral deficits at prodromal stages^14,17^.

In our previous work^14^, we characterized early behavioral deficits in TgAD rats and related them to measures of central neuroinflammation and AD-like pathology within brain regions critical for decision making, motivation, and executive function. The present study is a direct follow-up to that work, expanding the focus from intracerebral to systemic inflammation. Here, we apply principal component analysis and protein–protein interaction mapping to serum cytokine and growth factor data from the same TgAD and wild-type rats, constructing systemic inflammatory networks and examining their associations with cognitive performance and emotional affect. By integrating multivariate immune profiling with behavior, this study tests whether coordinated systemic immune activity is linked to early cognitive and motivational alterations in AD, providing new insight into peripheral contributors to disease-related dysfunction.

## Results

### Five serum inflammation profiles explain 65.6 percent of variance.

**Figure 1:**
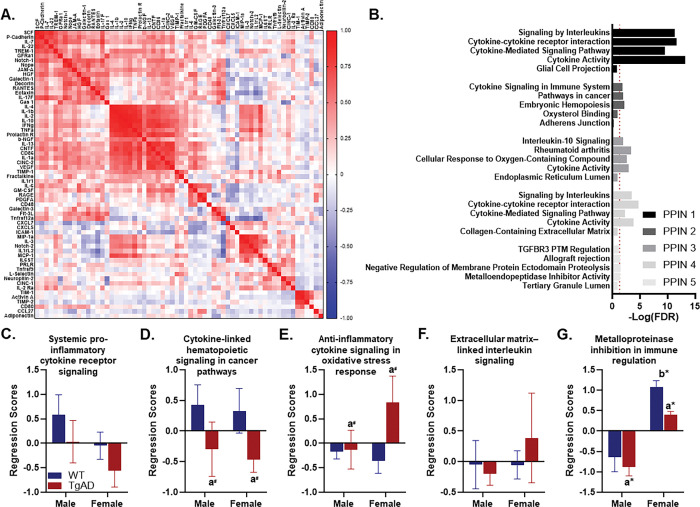
Serum inflammatory profiles altered by genotype and sex. Weighted protein correlation network in serum of WT and TgAD rats as determined by clustergrammer which validates PCA. Color gradient displays *r*-values. Red indicates values closer to r=1.0 (positive correlations), white indicates *r*-values near or equal to 0, and more blue values indicates values closer to *r*=−1.0 (negative correlations). **B.** EnrichR was used to determine significant (−LogFDR>1.3, denoted by dashed, red line) gene ontology terms for each cluster. The top terms are displayed. **C-G.** Multivariate ANOVA on PCA regression scores revealed group differences. In all panels, a*=*p*< 0.05 and a^#^=*p*< 0.1 for genotype main effects; b*=*p*< 0.05 for sex main effects. Dark blue = WT, dark red = TgAD. Group sizes: WT (n=8 females, 6 males), TgAD (n=6 females, 8 males).

To reduce dimensionality and identify biologically coherent profiles among serum inflammatory proteins, we conducted a PCA with Varimax rotation. Five components emerged with eigenvalues greater than 1.0 that together explained 65.55 percent of total variance. The rotated components accounted for 21.54, 15.75, 10.97, 9.59, and 7.70 percent of variance, respectively. Proteins were assigned to the component on which they had the highest absolute loading, so each protein contributed to only one profile (Fig. 1A).

Component 1 (systemic pro-inflammatory cytokine receptor signaling) was characterized by high loadings from proinflammatory cytokines including TNFα, IFNγ, IL-1β, IL-2, IL-4, IL-10, and IL-13, along with T-cell and monocyte activation markers such as CD86, IL-1α, MCP-1, and Fractalkine. Growth and neurotrophic factors (e.g., Beta-NGF, CNTF, PRLR) and matrix regulators (e.g., TIMP-1) were also represented, suggesting integration of immune signaling with neuroimmune crosstalk. Enrichment analysis using Enrichr identified highly significant overrepresentation of “Cytokine Activity,” “Cytokine–cytokine receptor interaction,” “Signaling by Interleukins,” and “Cytokine-mediated signaling pathway,” with all values well above the significance threshold (Fig. 1B). Weights derived from − log(FDR) scores prioritized these broad, high-magnitude cytokine receptor-mediated signaling processes, indicating a central role for interleukin-driven immune communication (Table S1).

Component 2 (cytokine-linked hematopoietic signaling in cancer pathways) comprised proteins involved in chemotaxis, extracellular matrix regulation, and tissue remodeling, including Eotaxin, RANTES, Decorin, FLT3 ligand, Galectin-1, and Gas1. Additional loadings from HGF, Nope, and Notch1 suggested engagement of stromal or progenitor cell signaling pathways. Enrichr analysis identified significant enrichment for “Embryonic hemopoiesis,” “Pathways in cancer,” and “Cytokine signaling in immune system,” indicating coordinated immune and hematopoietic processes with potential links to oncogenic signaling pathways (Fig. 1B). These weighted enrichments, combined with the chemokine-stromal signature reflected immune-driven tissue remodeling with stromal and progenitor cell involvement (Table S2).

Component 3 (anti-inflammatory cytokine signaling in oxidative stress response) included IL-6, ICAM-1, PDGF-AA, RAGE, L-selectin, CXCL5, and CCL27, markers strongly associated with endothelial activation, oxidative stress, and innate immune signaling. Enrichr analysis revealed significant enrichment for “Rheumatoid arthritis,” “Cytokine activity,” “Cellular response to oxygen-containing compound,” and “Interleukin-10 signaling,” suggesting a functional profile of cytokine-driven inflammatory signaling in the context of oxidative stress regulation (Fig. 1B). This pattern weighted toward anti-inflammatory IL-10–mediated pathways within an oxidative stress-responsive context (Table S3).

Component 4 (extracellular matrix-associated interleukin signaling) featured elevated loadings from IL-22, IL-17F, IL-7, and SCF, along with TREM-1 and P-cadherin. These proteins are associated with epithelial repair, mucosal immunity, and barrier protection, alongside amplification of innate immune responses. Enrichr analysis identified significant enrichment for “Cytokine–cytokine receptor interaction,” “Cytokine activity,” “Signaling by interleukins,” and “Cytokine-mediated signaling pathway,” along with a non-significant but contextually relevant “Collagen-containing extracellular matrix” term. While both Inflammation profiles 1 and 4 showed cytokine/interleukin enrichment, the lower magnitude enrichments here and the presence of ECM-related context suggested a more tissue-structural focus (Fig. 1B), emphasizing cytokine activity in tissue remodeling and barrier maintenance (Table S4).

Component 5 (metalloproteinase inhibition in immune regulation) was defined by a selective set of proteins, including Activin A, Neuropilin-2, Prolactin, TIM-1, CD80, and CINC1, associated with immune-endocrine signaling, developmental regulation, and tissue repair. Enrichr analysis revealed significant enrichment for “Negative regulation of membrane protein ectodomain proteolysis,” “Metalloendopeptidase inhibitor activity,” and “Allograft rejection,” indicating immune regulatory processes involving protease inhibition and immune activation (Fig. 1B). Integrating the enrichment profile with the modulatory repair functions of the protein set reflected its role in proteolytic control during immune responses and tissue repair (Table S5).

Together, these five components represent biologically distinct and functionally interpretable serum inflammation profiles, encompassing systemic pro-inflammatory signaling, hematopoietic and tissue remodeling pathways, oxidative stress-modulated anti-inflammatory signaling, extracellular matrix-associated interleukin activity, and metalloproteinase-regulated immune responses. These profiles may be differentially associated with genotype, sex, cognitive performance, and age-related pathology within the broader experimental dataset.

### Genotype and sex independently modulate systemic inflammation.

We next asked whether TgF344-AD genotype and sex influenced these systemic inflammation profiles. A multivariate general linear model with the five PCA-derived component regression scores as dependent variables revealed significant main effects of genotype (Wilks’ λ = 0.593, F_(5,20)_ = 2.746, p = 0.048, η_p_^2^=0.407) and sex (Wilks’ λ = 0.258, F_(5,20)_ = 11.492, p < 0.001, η_p_^2^=0.742). The genotype by sex interaction was not significant (Wilks’ λ = 0.849, F_(5,20)_ = 0.710, p = 0.623, η_p_^2^=0.151), indicating largely independent influences of genotype and sex at the multivariate level.

Follow-up univariate tests clarified which axes carried these effects. The systemic cytokine receptor signaling profile did not differ by genotype or sex and showed no interaction (Fs_(1,24)_ ≤ 2.675, ps > 0.11; Fig. 1C). The cytokine-linked hematopoietic signaling in cancer pathways profile showed a trend toward lower scores in TgAD rats relative to WT (F_(1,24)_ = 4.136, p = 0.053, η_p_^2^=0.147), with no sex effect or interaction (Fs_(1,24)_ ≤ 0.129, ps ≥ 0.723; Fig. 1D). The anti-inflammatory cytokine signaling in oxidative stress response profile trended toward higher scores in TgAD rats (F_(1,24)_ = 2.985, p = 0.097, η_p_^2^=0.111) without sex or interaction effects (Fs_(1,24)_ ≤ 2.563, ps ≥ 0.122; Fig. 1E). The extracellular matrix-associated interleukin signaling profile did not show significant main effects or interaction (Fs_(1,24)_ ≤ 0.544, ps ≥ 0.468; Fig. 1F). In contrast, the metalloproteinase inhibition in immune regulation profile exhibited clear group differences. Scores were lower in TgAD than in WT animals (F_(1,24)_ = 4.250, p = 0.050, η_p_^2^=0.150) and were markedly elevated in females regardless of genotype (F_(1,24)_ = 44.501, p < 0.001, η_p_^2^=0.650), with no interaction (Fig. 1G). In short, several profiles suggested subtle genotype-related shifts, but metalloproteinase-related immune regulation stood out as the most robustly altered by both genotype and sex.

While the follow-up univariate analyses established whether genotype and sex altered each inflammation profile as a singular entity, they did not reveal whether disease-related differences might emerge in the organization of proteins within a given profile. Because PCA aggregates proteins based on shared variance, profiles with similar overall scores can still differ substantially in how individual proteins relate to one another, a distinction that may be critical for understanding underlying biological mechanisms. We therefore examined each component as a protein–protein interaction network to determine whether TgAD altered network architecture within profiles.

### Profile networks.

#### Network 1: TgAD strengthens cytokine connectivity despite lower fractalkine.

**Figure 2:**
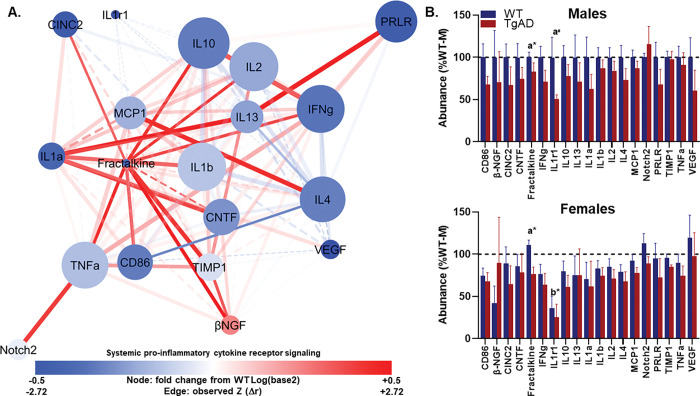
Systemic pro-inflammatory cytokine receptor signaling network *(Profile 1)*. **(A)** Protein–protein interaction network showing serum markers with the highest loadings on Component 1 from principal component analysis. Node size reflects absolute loading strength; node color indicates protein abundance (red = higher, blue = lower), and edge thickness denotes correlation strength between proteins. This network is enriched for interleukin-centered cytokine signaling pathways. **(B–C)** Mean (± SEM) protein abundances for each genotype (WT, blue; TgAD, red) in males **(B)** and females **(C)**. Nodes are scaled by loading score and color-coded by fold-change in TgAD relative to WT protein abundance (red=increase, blue=decrease). Edges represent protein-protein correlations, with thickness scaled to |*r*| (thickest near 1.0, thinnest near 0.0); line type indicating *p*-value (solid<0.1, dashed≥0.1), color denoting Z-score changes in correlation strength between WT and TgAD (red=positive change, blue=negative change), and transparency reflecting the *p*-value of the Z-score change (more opaque=more significant). In all panels, a*=*p*< 0.05 and a^#^=*p*< 0.1 for genotype main effects; b*=*p*< 0.05 for sex main effects. Dark blue = WT, dark red = TgAD. Group sizes: WT (n=8 females, 6 males), TgAD (n=6 females, 8 males).

Network 1 contained 19 proteins connected by 124 edges, forming a single, highly cohesive component. The average number of neighbors was 13.05, the clustering coefficient was 0.827, and network density was 0.725. Short characteristic path length (1.281) and a compact diameter and radius (3 and 2, respectively) indicated efficient connectivity. Network heterogeneity and centralization were modest (0.317 and 0.245), suggesting no single hub dominated global structure (Fig. 2A; Tables S6-S7; and all raw data is reported in Table S8).

In the TgAD group, 13 protein pairs demonstrated significant changes in correlations relative to WT. Among the most notable was the strong positive association between Fractalkine and bNGF (*r* = 0.713, p = 0.0042), which increased significantly from WT (Z = 2.72, p = 0.0033). Both proteins displayed high degree centrality (Fractalkine = 15; bNGF = 14) and moderate PCA loadings (0.434 and 0.577, respectively), suggesting central roles in network connectivity. IL13–PRLR (*r* = 0.941, p < 0.001; Z = 2.61, p = 0.0046) and IL13–IL1a (*r* = 0.921, p < 0.001; Z = 2.60, p = 0.0047) also showed pronounced increases in correlation, with IL13 exhibiting high centrality (degree = 15) and strong PCA loading (0.741). TNFa–Fractalkine (*r* = 0.662, p = 0.0099; Z = 2.36, p = 0.0092) and MCP1–IL4 (*r* = 0.918, p < 0.001; Z = 2.33, p = 0.0099) similarly increased, implicating coordinated changes across pro-inflammatory cytokines and chemokines.

Other significant increases included IL10–Fractalkine (*r* = 0.660, p = 0.010; Z = 2.27, p = 0.01), TIMP1–Fractalkine (*r* = 0.856, p < 0.001; Z = 2.24, p = 0.013), and Fractalkine–IL2 (*r* = 0.602, p = 0.023; Z = 2.20, p = 0.014), each involving Fractalkine as a recurrent hub. Notably, TNFa–Notch2 (*r* = 0.885, p < 0.001; Z = 2.12, p = 0.017) and CNTF–IL1a (*r* = 0.915, p < 0.001; Z = 2.03, p = 0.021) were among the strongest correlations overall, suggesting enhanced coupling between neurotrophic signaling and pro-inflammatory interleukins in TgAD. The remaining significant edges included PRLR–IL1a (*r* = 0.912, p < 0.001; Z = 1.99, p = 0.023), Notch2–IL1a (*r* = 0.911, p < 0.001; Z = 1.99, p = 0.023), and PRLR–Notch2 (*r* = 0.909, p < 0.001; Z = 1.98, p = 0.025). Collectively, these edges indicate that TgAD pathology is accompanied by strengthened co-regulation among key chemokines, interleukins, and neuroimmune modulators, with Fractalkine, IL13, and IL1a serving as high-centrality hubs.

An additional six protein pairs exhibited trending changes (TgAD correlation coefficient p ≤ 0.05, 0.05 ≤ p < 0.1 for *r* change or observed Z). These included CD86–Fractalkine (*r* = 0.684, p = 0.007; Z = 1.64, p = 0.0504), IL1b–IL1a (*r* = 0.817, p < 0.001; Z = 1.57, p = 0.058), and TIMP1–IL2 (*r* = 0.672, p = 0.008; Z = 1.44, p = 0.075), which may reflect coordinated changes in antigen presentation and cytokine signaling. TNFa–TIMP1 (*r* = 0.729, p = 0.003; Z = 1.40, p = 0.081), TNFa–IFNg (*r* = 0.908, p < 0.001; Z = 1.36, p = 0.087), and IL2–IL1a (*r* = 0.754, p = 0.002; Z = 1.30, p = 0.098) also trended toward increased correlation, reinforcing a broader pattern of enhanced connectivity between inflammatory and immune regulatory mediators in TgAD.

Given that proteins within each network were identified via principal component analysis and are likely to be biologically interrelated, we first conducted a MANOVA to assess the combined influence of genotype, sex, and their interaction on all proteins within each network. This approach was chosen to account for potential intercorrelations among proteins, control Type I error inflation from multiple univariate tests, and capture multivariate patterns that may not be detectable through single-protein analyses alone.

The multivariate analysis revealed a significant main effect of genotype on the combined abundance of Network 1 proteins (Wilks’ λ = 0.029, F_(19,6)_ = 10.691, p = 0.004, η_p_^2^=0.971), indicating that TgAD and WT animals differed in overall protein profiles within this network (Fig. 2B). A significant main effect of sex was also detected (Wilks’ λ = 0.043, F_(19,6)_ = 7.024, p = 0.012, η_p_^2^=0.957), suggesting that males and females differed in multivariate protein abundance patterns. The genotype × sex interaction was not significant (Wilks’ λ = 0.253, F_(19,6)_ = 0.932, p 0.587, η_p_^2^=0.747). Follow-up univariate tests identified significant genotype effects for Fractalkine with a lower serum concentrations in TgAD rats (F_(1,24)_ = 9.591, p = 0.005, η_p_^2^=0.286) and a trend toward a lower serum concentration in TgAD rats for IL1r1 (F_(1,24)_ = 3.959, p = 0.058, η_p_^2^=0.142) such that there was lower abundance in TgAD rats relative to WT. Sex differences were significant for IL1r1 (F_(1,24)_ = 8.707, p = 0.007, η_p_^2^=0.266) such that females showed lower protein abundance (Fig. 2B), and no other proteins reached significance for sex. No individual proteins showed significant genotype × sex interaction effects at the univariate level.

#### Network 2: Stromal and progenitor signaling proteins are reduced in TgAD.

**Figure 3:**
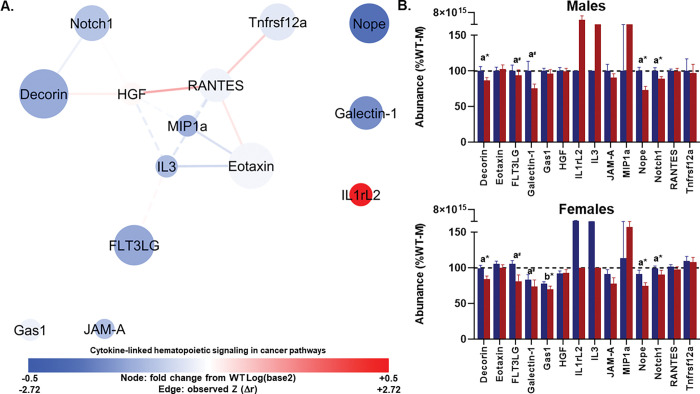
Cytokine-linked hematopoietic signaling in cancer pathways network (Profile 2). **(A)** Protein–protein interaction network showing serum markers with the highest loadings on Component 2 from principal component analysis. Node size reflects absolute loading strength; node color indicates relative protein abundance (red = higher, blue = lower), and edge thickness denotes correlation strength between proteins. This network is enriched for immune-related hematopoietic signaling with links to chemokine-mediated cell recruitment. **(B–C)** Mean (± SEM) protein abundances for each genotype (WT, blue; TgAD, red) in males **(B)** and females **(C)**. Nodes are scaled by loading score and color-coded by fold-change in TgAD relative to WT protein abundance (red=increase, blue=decrease). Edges represent protein-protein correlations, with thickness scaled to |*r*| (thickest near 1.0, thinnest near 0.0); line type indicating *p*-value (solid<0.1, dashed≥0.1), color denoting Z-score changes in correlation strength between WT and TgAD (red=positive change, blue=negative change), and transparency reflecting the *p*-value of the Z-score change (more opaque=more significant). In all panels, a*=*p*< 0.05 and a^#^=*p*< 0.1 for genotype main effects; b*=*p*< 0.05 for sex main effects. Dark blue = WT, dark red = TgAD. Group sizes: WT (n=8 females, 6 males), TgAD (n=6 females, 8 males).

Network 2 consisted of 14 nodes and 14 edges, with an average of 3.11 neighbors per node. Topology was more fragmented than Network 1, with six connected components, a moderate clustering coefficient (0.581), and a lower density of 0.389. The path structure was still efficient, with a diameter of 3, radius of 2, and characteristic path length of 1.81, while heterogeneity and centralization were moderate (0.513 and 0.304), consistent with a few relatively influential nodes within a sparser graph (Fig. 3A; Tables S6-S7).

No edges met the primary criterion for significance. One edge met the trend criterion. HGF–RANTES showed a positive association in TgAD (*r* = 0.690, p = 0.006), with an observed Z-change = 1.376 and a trend toward significance (p = 0.084) relative to WT. Node attributes for these proteins in this component indicate moderate PCA loadings (HGF loading = 0.581; RANTES loading = 0.690), comparable degrees (HGF degree = 5; RANTES degree = 5), and modest betweenness (HGF = 0.429; RANTES = 0.286). Consistent with node encodings, HGF showed a small positive fold change (+ 0.014), whereas RANTES showed a small negative fold change (− 0.031).

The MANOVA revealed significant multivariate effects of genotype (Wilks’ λ = 0.103, F_(14,11)_ = 6.806, p = 0.001, η_p_^2^=0.897) and sex (Wilks’ λ = 0.082, F_(14,11)_ = 8.790, p < 0.001, η_p_^2^=0.918) on the collective protein profile (Fig. 3B). The genotype × sex interaction was not significant (Wilks’ λ = 0.418, F_(14,11)_ = 1.094, p = 0.448, η_p_^2^=0.582). Follow-up univariate ANOVAs identified that protein serum concentration was significantly lower in TgAD rats for Decorin (F_(1,24)_ = 8.927, p = 0.006, η_p_^2^=0.271), Nope (F_(1,24)_ = 16.821, p < 0.001, η_p_^2^=0.412), and Notch1 (F_(1,24)_ = 5.618, p = 0.026, η_p_^2^=0.190), with trends for FLT3LG (F_(1,24)_ = 4.125, p = 0.053) and Galectin-1 (F_(1,24)_ = 3.707, p = 0.066; Fig. 3B). In female rats, Gas1 was significantly lower (F_(1,24)_ = 27.439, p < 0.001, η_p_^2^=0.533), with no other proteins reaching significance. No significant genotype × sex interaction effects emerged for any protein.

#### Network 3 : IL-6 hub relationships are selectively remodeled in TgAD.

**Figure 4:**
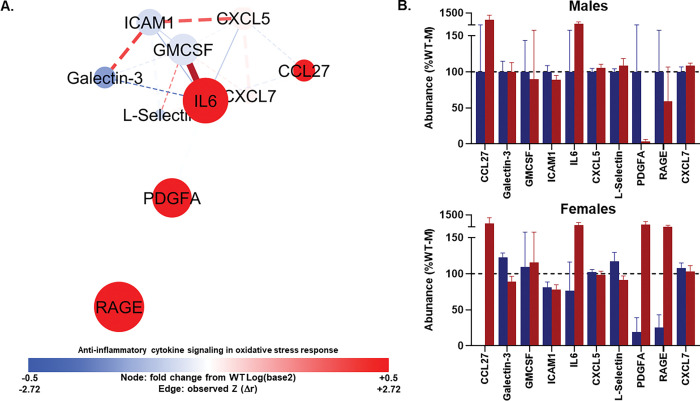
Anti-inflammatory cytokine signaling in oxidative stress response network (Profile 3). **(A)** Protein–protein interaction network showing serum markers with the highest loadings on Component 3 from principal component analysis. Node size reflects absolute loading strength; node color indicates relative protein abundance (red = higher, blue = lower), and edge thickness denotes correlation strength between proteins. This network is enriched for IL-6–driven acute-phase responses and chemokine-mediated leukocyte adhesion/migration. **(B–C)** Mean (± SEM) protein abundances for each genotype (WT, blue; TgAD, red) in males **(B)** and females **(C)**. Nodes are scaled by loading score and color-coded by fold-change in TgAD relative to WT protein abundance (red=increase, blue=decrease). Edges represent protein-protein correlations, with thickness scaled to |*r*| (thickest near 1.0, thinnest near 0.0); line type indicating *p*-value (solid<0.1, dashed≥0.1), color denoting Z-score changes in correlation strength between WT and TgAD (red=positive change, blue=negative change), and transparency reflecting the *p*-value of the Z-score change (more opaque=more significant). In all panels, a*=*p*< 0.05 and a^#^=*p*< 0.1 for genotype main effects; b*=*p*< 0.05 for sex main effects. Dark blue = WT, dark red = TgAD. Group sizes: WT (n=8 females, 6 males), TgAD (n=6 females, 8 males).

Network 3 comprised 10 nodes and 21 edges, a compact architecture with strong local clustering (clustering coefficient 0.759), density of 0.583, and short characteristic path length of 1.42. The diameter and radius were 2 and 1, and centralization was relatively high at 0.536, indicating that a subset of nodes served as hubs. The network split into two connected components, one large and one small (Fig. 4A; Tables S6-S7).

In the TgAD group, one protein pair exhibited a significant change in correlation relative to WT, and two pairs demonstrated trending changes. The most pronounced shift was observed between IL6 and GM-CSF, which showed a very strong positive correlation in TgAD (*r* = 0.928, p < 0.001) that was significantly stronger than in WT (Z = 2.9137, p = 0.002). IL6 displayed a high PCA loading (0.7307), a large positive fold change (+ 2.0561), and high centrality (degree = 8, betweenness = 0.304), while GM-CSF also showed a strong PCA loading (0.6109) and moderate network integration (degree = 6, betweenness = 0.0595), despite a small negative fold change (− 0.0673).

Two additional edges exhibited trending changes in correlation. CXCL5 and IL6 were strongly and significantly correlated in TgAD (*r* = 0.6210, p = 0.018) with a positive Z-change (1.3172, p = 0.093) relative to WT. CXCL5 had a negative PCA loading (− 0.545) and minimal fold change (+ 0.023), with modest connectivity (degree = 5, betweenness = 0.039), whereas IL6 again emerged as a highly central, high-loading node. In contrast, IL6 and ICAM1 demonstrated a significant negative correlation in TgAD (*r* = − 0.630, p = 0.016) with a negative Z-change (− 1.381, p = 0.084) compared to WT. ICAM1 exhibited a negative PCA loading (− 0.568), a slight negative fold change (− 0.088), and moderate degree (5) with low betweenness (0.033). Together, these results highlight IL6 as a central hub in inflammation profile network 3, with altered associations to both pro-inflammatory and adhesion-related factors in TgAD.

Despite these focal edge changes, the MANOVA revealed no significant overall multivariate effects of genotype, sex, or an interaction (Wilks’ λ ≥ 0.510, Fs_(1015)_ ≤ 1.444, ps ≥ 0.252).

#### Network 4: Barrier cytokines show tighter coupling in TgAD.

**Figure 5:**
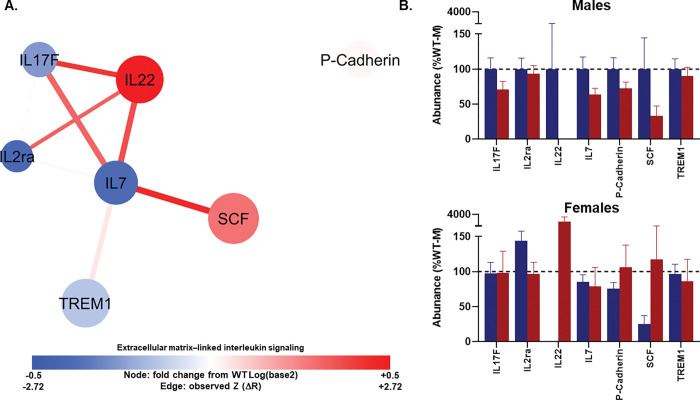
Extracellular matrix-linked interleukin signaling network (Profile 4). **(A)** Protein–protein interaction network showing serum markers with the highest loadings on profile 4 from principal component analysis. Node size reflects loading strength; node color indicates relative protein abundance (red = higher, blue = lower), and edge thickness denotes correlation strength between proteins. This network is enriched for IL-7 family cytokine signaling, including IL-17F and IL-22–associated pathways. **(B–C)** Mean (± SEM) protein abundances for each genotype (WT, blue; TgAD, red) in males **(B)** and females **(C)**. Nodes are scaled by loading score and color-coded by fold-change in TgAD relative to WT protein abundance (red=increase, blue=decrease). Edges represent protein-protein correlations, with thickness scaled to |*r*| (thickest near 1.0, thinnest near 0.0); line type indicating *p*-value (solid<0.1, dashed≥0.1), color denoting Z-score changes in correlation strength between WT and TgAD (red=positive change, blue=negative change), and transparency reflecting the *p*-value of the Z-score change (more opaque=more significant). In all panels, a*=*p*< 0.05 and a^#^=*p*< 0.1 for genotype main effects; b*=*p*< 0.05 for sex main effects. Dark blue = WT, dark red = TgAD. Group sizes: WT (n=8 females, 6 males), TgAD (n=6 females, 8 males).

Network 4 had 7 nodes and 8 edges. The average number of neighbors was 2.67, characteristic path length was short at 1.47, and the clustering coefficient was 0.550. Density was 0.533, heterogeneity 0.515, and centralization was relatively high at 0.700, suggesting a topology with a small number of influential nodes. The network comprised two connected components, a primary cluster and a smaller subgroup (Fig. 5A; Tables S6-S7).

In the TgAD group, three protein pairs exhibited significant changes in correlations relative to WT, with an additional two pairs showing trending changes. The strongest shift was observed between SCF and IL7, which demonstrated a very strong positive correlation in TgAD (*r* = 0.938, p < 0.001) that was significantly greater than in WT (Z = 2.416, p = 0.008). SCF exhibited a high PCA loading (0.802), a modest positive fold change (+ 0.275), and low network connectivity (degree = 1, betweenness = 0), whereas IL7 showed a similarly high PCA loading (0.739) and greater connectivity (degree = 5, betweenness = 0.7), alongside a negative fold change (− 0.388). Another strong correlation shift was found between IL22 and IL17F (*r* = 0.776, p = 0.001; Z = 2.1988, p = 0.014), with both proteins showing high PCA loadings (0.773 and 0.660, respectively) and moderate degrees (3 each), but in opposite fold-change directions (IL22 = + 3.977; IL17F = − 0.256). A third significant change occurred between IL22 and IL7 (*r* = 0.887, p < 0.001; Z = 1.8881, p = 0.030), linking two high-loading nodes in a strengthened positive association in TgAD.

Two additional edges exhibited trending changes in correlation. IL22 and IL2ra were moderately and significantly correlated in TgAD (*r* = 0.593, p = 0.025) with a positive Z-change (1.644, p = 0.050), connecting a strongly upregulated IL22 (+ 3.977) to a high-loading IL2ra (0.633) with a negative fold change (− 0.398). IL7 and IL17F showed a strong positive correlation (*r* = 0.882, p < 0.001) with a positive Z-change (1.6082, p = 0.054), linking two cytokines with high PCA loadings and moderate network connectivity. Collectively, these findings identify IL22, IL7, and IL17F as recurrently involved in altered correlation patterns, with IL22 appearing in three of the five altered edges.

At the multivariate level, there were no significant effects of genotype or sex on mean abundance of proteins in Network 4 and no interaction within Network 4 (Wilks’ λ ≥ 0.613, Fs_(1015)_ ≤ 1.625, ps ≥ 0.192).

#### Network 5: Sparse architecture with strong genotype and sex effects.

**Figure 6:**
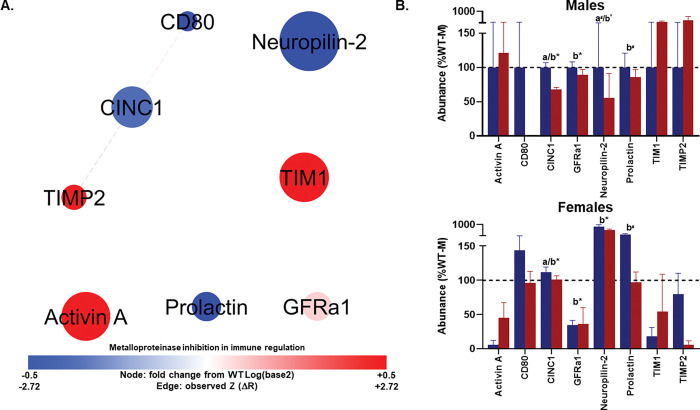
Metalloproteinase inhibition in immune regulation network (Profile 5). **(A)** Protein–protein interaction network showing serum markers with the highest loadings on profile 5 from principal component analysis. Node size reflects absolute loading strength; node color indicates relative protein abundance (red = higher, blue = lower), and edge thickness denotes correlation strength between proteins. This network is enriched for signaling through membrane-bound receptors and extracellular matrix–associated proteins, including TIM family members and Activin A. **(B–C)** Mean (± SEM) protein abundances for each genotype (WT, blue; TgAD, red) in males **(B)** and females **(C)**. Nodes are scaled by loading score and color-coded by fold-change in TgAD relative to WT protein abundance (red=increase, blue=decrease). Edges represent protein-protein correlations, with thickness scaled to |*r*| (thickest near 1.0, thinnest near 0.0); line type indicating *p*-value (solid<0.1, dashed≥0.1), color denoting Z-score changes in correlation strength between WT and TgAD (red=positive change, blue=negative change), and transparency reflecting the *p*-value of the Z-score change (more opaque=more significant). In all panels, a*=*p*< 0.05 and a^#^=*p*< 0.1 for genotype main effects; b*=*p*< 0.05 for sex main effects. Dark blue = WT, dark red = TgAD. Group sizes: WT (n=8 females, 6 males), TgAD (n=6 females, 8 males).

Network 5 contained 8 nodes and only 2 edges. The characteristic path length among connected nodes was 1.33, diameter 2, and radius 1. Local clustering was absent, density was relatively high because of the small node count, heterogeneity modest, and centralization maximal, consistent with a topology dominated by a single central connection amid otherwise isolated nodes. The graph contained six connected components, reflecting fragmentation at the pairwise level (Fig. 6A; Tables S6-S7).

For inflammation profile network 5, no protein pairs met the joint criteria of a significant or trending change in correlation between WT and TgAD and a significant correlation in TgAD (p ≤ 0.05). As such, no edges within this network qualified for further interpretation under the current analytical thresholds.

Despite the sparse edge structure, the MANOVA revealed significant multivariate effects of genotype (Wilks’ λ = 0.440, F_(8,17)_ = 2.704, p = 0.040, η_p_^2^=0.560; Fig. 6B). There was also a significant main effect of sex (Wilks’ λ = 0.146, F_(8,17)_ = 12.445, p < 0.001, η_p_^2^ = 0.854). The genotype × sex interaction was not statistically significant (p = 0.249). Follow-up univariate tests indicated that the main effect of genotype was driven by differences in CINC1 (F_(1,24)_ = 11.832, p = 0.002, η_p_^2^=0.330), with TgAD rats exhibiting lower serum concentrations relative to WT. Prolactin (F_(1,24)_ = 3.699, p = 0.066, η_p_^2^=0.134) and Neuropilin-2 (F_(1, 24)_ = 2.908, p = 0.10, η_p_^2^=0.108) showed trends toward lower TgAD serum concentrations (Fig. 6B). The main effect of sex was significant for CINC1 (F_(1,24)_ = 12.689, p = 0.002, η_p_^2^=0.346), GFRα1 (F_(1,24)_ = 22.851, p < 0.001, η_p_^2^=0.488), and Neuropilin-2 (F_(1,24)_ = 54.388, p < 0.001, η_p_^2^=0.694). Prolactin exhibited a trend toward sex differences (F_(1,24)_ = 3.434, p = 0.076, η_p_^2^=0.125). No proteins demonstrated significant genotype × sex interactions.

#### Maladaptive intertemporal choice is predicted by Network 4 trends.

We next evaluated whether these inflammatory axes explained unique variance in decision making, above and beyond sex and genotype. In a hierarchical model predicting average choice across 10–60 s delays on the intertemporal choice task, Model 1 with sex and genotype was significant (F_(2,25)_ = 5.75, p = 0.009), accounting for 31.5 percent of variance (R^2^=0.315, adjusted R^2^=0.260). Genotype was a significant predictor (B = 26.84, β = 0.567, t = 3.39, p = 0.002), consistent with more maladaptive choice in TgAD; sex was not significant (p = 0.627).

**Figure 7:**
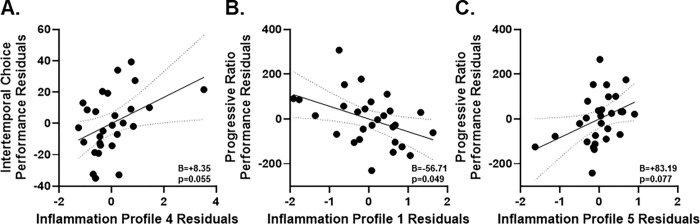
Behavioral performance associations with serum inflammation profiles after controlling for genotype and sex. **(A)** Intertemporal choice performance residuals plotted against residuals for Profile 4 (Extracellular matrix–linked interleukin signaling) scores, illustrating the positive relationship between maladaptive choice (driven by TgAD rats) and greater profile 4 scores. **(B)** Progressive ratio performance residuals plotted against residuals for Profile 1 (Systemic pro-inflammatory cytokine receptor signaling) scores, reflecting the negative association between motivation for reward and systemic pro-inflammatory cytokine receptor signaling. **(C)** Progressive ratio performance residuals plotted against residuals for profile 5 (Metalloproteinase inhibition in immune regulation) scores, highlighting links between reward motivation and cell surface receptor/extracellular matrix signaling.

Adding the five serum components in Model 2 increased total explained variance to 46.5 percent (R^2^=0.465, adjusted R^2^=0.278) and the overall model approached significance (F_(7,20)_ = 2.49, p = 0.052), but the change in R^2^ was not significant (ΔR^2^=0.150, F-change_(5,20)_ = 1.13, p = 0.379). Genotype remained significant (B = 26.74, β = 0.565, t = 2.68, p = 0.014). Among the components, Profile 4 showed a trend toward association with greater preference for delayed rewards (B = 8.35, β = 0.347, t = 2.04, p = 0.055; Fig. 7A), and other components were not significant predictors (ps = 0.187–0.918). Collinearity diagnostics were acceptable, with all VIFs less than 4 except Profile 5 at 3.718, which approached the threshold. Overall, genotype was the dominant predictor of decision strategy, with a suggestive contribution from the extracellular matrix-associated interleukin profile.

#### Motivation decreases with Network 1, and trends upward with Network 5.

To assess associations with motivated behavior, we modeled progressive ratio lever presses. In Step 1, sex and genotype explained a significant portion of variance (R^2^=0.396, adjusted R^2^=0.348; F(2,25) = 8.212, p = 0.002), with higher performance in females and in WT animals (sex: B = 117.312, β = 0.365, p = 0.029; genotype: B=−149.313, β=−0.464, p = 0.007).

In Step 2, adding the five serum components yielded a significant overall model (F_(7,20)_ = 4.298, p = 0.005) with R^2^=0.601 (adjusted R^2^=0.461), but the change in R^2^ was not significant (ΔR^2^=0.204, ΔF_(5,20)_ = 2.045, p = 0.116). Importantly, Profile 1 significantly predicted lower lever pressing (B = − 56.705, β=−0.346, t = − 2.093, p = 0.049; Fig. 7B), consistent with a link between systemic cytokine receptor signaling and reduced motivation. Profile 5 trended toward predicting higher lever pressing (B = 83.19, β = 0.508, t = 1.863, p = 0.077; Fig. 7C). With components in the model, genotype dropped to trend level (B = − 112.310, β=−0.349, t = − 1.915, p = 0.070) and sex was no longer significant (β=−0.075, p = 0.791), suggesting that inflammatory profiles, particularly Profile 1, carry unique variance related to apathy-like behavior.

#### Set-shifting shows no association with inflammation profiles.

We examined whether serum profiles related to set-shifting performance indexed by trials to criterion. Sex and genotype alone explained negligible variance (R^2^=0.009, F_(2,25)_ = 0.110, p = 0.896). Adding the five components increased total explained variance to 28.3 percent (adjusted R^2^=0.032), but the increment was not significant (ΔR^2^=0.274, F_(5,20)_ = 1.529, p = 0.226). Thus, under these task conditions, systemic inflammatory axes did not meaningfully account for individual differences in set-shifting beyond genotype and sex.

#### Working memory deficits reflect genotype rather than inflammation profiles.

Finally, we asked whether inflammatory profiles were associated with delayed matching-to-sample accuracy at the 18–24 s delay. Model 1 indicated that genotype and sex together accounted for a significant portion of variance (R^2^=0.371, adjusted R^2^=0.320, F_(2,25)_ = 7.361, p = 0.003). TgAD animals performed worse than WT (B = − 8.438, β=−0.479, t = − 2.986, p = 0.006). Sex showed a trend toward better performance in females (B = 5.536, β = 0.314, t = 1.959, p = 0.061).

Adding the five components in Model 2 produced a significant overall model (F_(7,20)_ = 2.697, p = 0.039) with R^2^=0.486 (adjusted R^2^=0.305), but the increment in explained variance was not significant (ΔR^2^=0.115, ΔF_(5,20)_ = 0.893, p = 0.504). No serum component was a significant predictor. Genotype remained significant in the expanded model (B = − 7.588, β=−0.430, t = − 2.081, p = 0.050), whereas sex was not (p = 0.135). Diagnostics indicated acceptable multicollinearity and no autocorrelated residuals. These findings indicate that working memory deficits at longer delays are primarily captured by genotype rather than by circulating inflammatory profiles.

## Discussion

This study identified five biologically distinct serum inflammatory profiles in the TgAD rat model and demonstrated that genotype and sex exert strong yet largely independent effects on these immune profiles. Principal component analysis reduced the complex cytokine dataset into interpretable inflammatory profiles, which were further characterized through network analyses. By extending protein–protein interaction (PPI) and module-mapping approaches previously used in human plasma^5^ and in brain tissue from mouse AD models^6–8^ into the serum compartment of an AD model, we address a major gap in systemic network biology of Alzheimer’s disease. Genotype effects were most pronounced in profiles dominated by classical pro-inflammatory cytokines and in networks centered on chemokines and neuroimmune modulators, whereas sex effects reflected a female bias toward elevated pro-inflammatory signaling. Several of these inflammatory signatures were also linked to discrete cognitive and motivational domains, suggesting functional specificity in the relationship between systemic immune states and behavior.

Genotype effects were evident at both the profile and network levels. TgAD rats exhibited significantly higher scores on Profile 5, comprising classical pro-inflammatory cytokines and immune-endocrine regulators, consistent with clinical reports that AD is associated with heightened systemic inflammation^2,18^. Network analyses revealed more nuanced disease-related restructuring. In Network 1, TgAD rats displayed strengthened co-regulation among Fractalkine, IL-13, IL-1α, and related interleukins, with Fractalkine emerging as a high-centrality hub. Given Fractalkine’s role in neuron–microglia signaling^19–21^, such peripheral network changes may parallel or influence central neuroimmune activation. In Network 2, TgAD rats showed reduced abundance of extracellular matrix- and progenitor-associated markers, including Decorin, Nope, and Notch1, suggesting impaired tissue remodeling and stromal signaling^22–25^. Notably, although many proteins in Networks 1 and 2, including Fractalkine and IL1r1, were less abundant in TgAD, their correlations were stronger than in WT. This pattern is consistent with findings from human plasma proteomics^5^, where several protein modules showed reduced abundance in AD patients compared to cognitively healthy controls. While the specific proteins differ, both studies indicate that coordinated immune modules, rather than isolated proteins, are altered in AD. In both species, such reductions occur at the level of coordinated, biologically coherent modules rather than isolated proteins, reinforcing the concept of AD as a disorder of systemic, network-wide immune dysregulation.

Mechanistically, reduced abundance within tightly defined inflammatory networks may reflect suppressed trophic or immune signaling, altered protein turnover, or compensatory recalibration in response to chronic pathology. Our additional finding that network-wide correlations tightened despite reduced abundance suggests a genotype-linked shift toward more rigid coordination among remaining proteins. From a systems biology perspective, this tightening is consistent with loss-of-complexity models of aging and disease, in which normally semi-independent physiological subsystems become more tightly coupled, reducing variability, adaptability, and resilience^26–28^. Network theory further predicts that such increases in correlation coherence can signal approach to a critical transition, where a subset of variables becomes highly synchronized, a dynamic network biomarker of reduced resilience^29–31^. In immune systems, this has been interpreted as dominance of shared upstream drivers, potentially constraining adaptive responses while sustaining a chronic, organized inflammatory state^32,33^.

Sex differences in serum inflammatory profiles were robust, largely stemming from higher profile 5 scores in females across genotypes. This aligns with well-established immune dimorphism, wherein females mount stronger innate and adaptive immune responses^34,35^ and may contribute to the greater incidence and accelerated progression of Alzheimer’s disease in women. In Network 2, female rats also showed lower abundance of Gas1, a growth arrest–associated marker, compared to males. Because Gas1 can influence cell survival and differentiation cues, a reduced abundance in females might reflect sex-specific variation in peripheral tissue remodeling or repair processes. Indeed, Gas1 can suppress responsiveness to ovulatory signals, and its depletion enhances tissue remodeling gene expression in female reproductive tissues^36^. However, our network correlations were computed within genotype without stratifying by sex, and thus we cannot infer sex-specific differences in co-regulation from the present data. Nevertheless, in principle, tighter cytokine co-fluctuation has been linked to reduced network flexibility and to pre-transition dynamics in other physiological and immunological contexts^26,28,29,32^, underscoring the value of future sex-stratified network analyses.

Network reorganization analyses revealed that genotype effects often manifested as changes in correlation structure rather than absolute abundance. In Network 3, IL-6 emerged as a central hub with altered associations to both pro-inflammatory (GM-CSF, CXCL5) and adhesion-related (ICAM-1) factors, suggesting that TgAD pathology may shift IL-6’s functional role from coordinated repair responses toward maladaptive pro-inflammatory signaling. In Network 4, IL-22, a cytokine involved in epithelial barrier maintenance, appeared in multiple altered correlations, raising the possibility that barrier-protective pathways are differentially regulated in TgAD. Such restructuring of inflammatory coordination could alter the temporal dynamics of immune responses, thereby impacting downstream neuroimmune interactions.

Behavioral associations demonstrated that inflammatory profiles were differentially related to distinct cognitive and motivational domains. Profile 1 (systemic proinflammatory cytokine receptor signaling) was independently associated with reduced motivation on the progressiveratio task, consistent with evidence that inflammation disrupts corticostriatal reward circuitry and dopamine signaling, contributing to anhedonia and motivational impairment^37^. Profile 4 (extracellular matrix–associated interleukin signaling) trended toward association with maladaptive choice in the intertemporal decision-making task, a finding that may reflect the influence of ECM and cytokine-driven modulation on neural circuits governing value-based choice. ECM structures such as perineuronal nets regulate synaptic plasticity and behavioral flexibility in prefrontal regions^38^, while interleukins such as IL-1, IL-6, and IL-17 can impair synaptic plasticity and cognitive processing^39,40^. In addition, inflammatory signaling has been shown to bias decision-making toward maladaptive patterns, including increased sensitivity to punishment over reward and steeper delay discounting^41–43^. As reported in our previous study^14^, working memory performance at longer delays was driven by genotype rather than serum inflammation, suggesting that in this domain, and within the context of this study, central neuropathology may overshadow peripheral immune contributions.

This study builds directly on our prior report^14^ in which we characterized Alzheimer’s-like pathology in young adult TgF344-AD rats as producing neuroinflammatory changes within a prefrontal-amygdala-striatal circuit and corresponding deficits in motivation and decision-making. In that work, impairments on the intertemporal choice task (decision-making), progressive ratio task (motivation), set-shifting task (cognitive flexibility), and delayed matching-to-sample task (working memory) were presented alongside central cytokine/chemokine alterations, establishing a circuit-level neuroimmune basis for these behavioral changes. Here, we re-utilize the same behavioral dataset to examine their associations with newly characterized systemic inflammatory profiles derived from principal component and network analyses of serum proteins. This approach reveals that the same behavioral phenotypes previously linked to central neuroinflammation also align with distinct peripheral inflammatory architectures, including pro-inflammatory cytokine dominance, extracellular matrix-associated interleukin signaling, and altered chemokine-growth factor coordination. Thus, while the behavioral outcomes themselves are unchanged from our earlier publication, their integration with the present serum analyses demonstrates a convergence between central and systemic immune network changes in early Alzheimer’s-like pathology and identifies peripheral immune signatures that may reflect or influence the neuroimmune mechanisms underlying cognitive and motivational dysfunction.

## Conclusions

In summary, these findings demonstrate that Alzheimer’s-like pathology in the TgF344-AD rat is associated with both quantitative and topological changes in systemic inflammatory networks, and that these changes are functionally relevant to specific behavioral outcomes. The dissociation of effects across inflammatory components underscores that distinct immune profiles, rather than generalized inflammation, may differentially contribute to motivational, decision-making, and cognitive control processes. Limitations include the cross-sectional design, reliance on serum measures without direct CNS correlates, and modest sample size. Future work should employ longitudinal designs to determine the temporal relationships between peripheral inflammation, network restructuring, and cognitive decline, as well as integrate central immune profiling to better understand the mechanistic links between systemic and neuroinflammatory processes. Such approaches may clarify whether specific inflammatory profiles, particularly those dominated by classical pro-inflammatory cytokines and systemic cytokine receptor signaling, could serve as biomarkers or therapeutic targets in Alzheimer’s disease.

## Materials and Methods

### Subjects.

A total of 28 wild type (WT) and TgAD rats were used for behavioral and neurobiological experiments (WT: n = 8 Female, n = 6 Male; TgAD: n = 8 Male, n = 6 Female). As previously described^14^, TgAD rats containing the human Swedish mutation amyloid precursor protein (APP^swe^) and the presenilin-1 exon 9 deletion mutant (PS1^ΔE9^) were bred with WT F344 females (Envigo: previously Harlan Laboratories) at the University of Alabama at Birmingham. All breeding, experimental procedures, and animal endpoints were approved by the University of Alabama Institutional Animal Care and Use Committee and follow guidelines set by the National Institutes of Health. This study is reported in accordance with the ARRIVE guidelines (Animal Research: Reporting of In Vivo Experiments, 2020 revision). The original breeding pair was obtained from University of Southern California, Los Angeles, CA^16^. Rats were maintained under standard animal care facility conditions with food (catalog #Harlan 2916, Teklad Diets) and water *ad libitum* and a 12 h reverse light/dark cycle (lights off at 7am) at 22°C and 50% humidity. Rats were housed in standard rat cages (height, 7 inches; floor area, 144 square inches) in same-sex groups of four or less at weights of ~300 g or two per cage when ≥ 400 g. Rats were aged from birth to experimental age groups categorized as young adults during prodromal stages. On average, rats were 9.4 months old at the time of sacrifice for cytokine, chemokine, immunotropic factor analyses.

### Blood serum measures.

#### Tissue preparation.

At the end of behavioral testing, rats were given a two-week period in which they were returned to ad libitum feeding prior to blood serum collection. Procedures were done according to our previous work^44^. Blood serum was collected from rats a minimum of two weeks after the final operant task. In accordance with UAB IACUC approval, rats were briefly restrained via decapicone (Braintree Scientific), rapidly decapitated as reported in many of our prior studies^14,45–50^, and trunk blood was collected into individual 1.7mL tubes. Tubes were then incubated on ice for a minimum of 20 minutes prior to plasma-serum separation via centrifugation.

#### Multiplex ELISA.

At least 100μL of serum from each sample was assessed by multiplex ELISA (Raybiotech) using arrays focused on proteins associated with inflammation (Rat Cytokine Array Q67; see Supplementary Table S9 for Gene-Protein names and descriptions). Each animal served as a biological replicate, and as such, there were n = 14 WT and n = 14 TgAD samples.

#### Behavioral Performance.

Behavioral testing data (progressive ratio, intertemporal choice, set-shifting, and delayed matching-to-sample tasks) were collected and fully reported in Hernandez et al., 2024; the same dataset is reanalyzed here exclusively to examine its associations with newly derived serum inflammatory profiles. Briefly, rats were trained and tested in operant conditioning chambers (Coulbourn Instruments) equipped with two retractable levers, a central food trough, and associated cue and house lights. Animals completed a battery of operant-based cognitive and motivational tasks, including a delay discounting (intertemporal choice) task to assess preference for a small immediate reward versus a larger delayed reward with delays increasing across trial blocks (0–60 s), a progressive ratio task to measure motivation by increasing the number of lever presses required to obtain a reward until animals ceased responding (breakpoint), a set shifting task to evaluate cognitive flexibility by requiring a shift from a visual cue–based rule to a spatial lever position–based rule, and a delayed response task to assess working memory by requiring correct lever selection following variable delays between sample and choice phases. In the present study, these previously published behavioral data were used solely as dependent measures to examine their relationships with serum inflammatory marker networks, genotype (TgAD vs. WT), and sex.

### Statistical analysis and experimental design.

#### General statistical approach.

Unless otherwise noted, all statistical analyses were performed in SPPSS28 v280.0.0(190). In all analyses, the alpha (α) was set to 0.05. When there were significant effects, the effect sizes were reported as partial η^2^ (η_p_^2^) for ANOVAs. For brevity, null effects were reported as consolidated F- or t-statistics, p-values, and only the lowest and highest values for each were given. All figures were generated in GraphPad Prism v10.4.2(633). All data generated or analyzed during this study are included in this published article.

#### Network Construction and Analysis.

We adapted a modified version of the analysis pipeline described by Elkhatib et al. (2020) to reduce the dimensionality of multiplex ELISA data^51^. Protein concentrations from each sample were subjected to principal component analysis (PCA) with Varimax rotation and Kaiser normalization in IBM SPSS Statistics (Version 29). The number of components was determined using the Scree method, and component scores were extracted as regression loadings. Proteins were assigned to components based on their highest absolute factor loading (≥ 0.4), with each component representing a distinct inflammatory protein profile. To validate PCA-derived components, we performed weighted correlation network analysis in Clustergrammer^52^, enabling visualization and quantification of interrelationships among serum inflammatory markers.

To assign biologically meaningful labels to inflammatory protein profiles, we used the Enrichr platform to perform enrichment analyses against the Gene Ontology (GO) Biological Process, GO Molecular Function, GO Cellular Component, KEGG Pathway^53,54^, and Reactome Pathway databases. For each inflammatory profiles, the list of proteins with the highest loadings was submitted to Enrichr, and the top enriched terms from each database were extracted along with their corresponding false discovery rate (FDR5%) values. The FDR values were converted to − log(FDR_5%_) scores, with 1.3 as the statistical significance threshold, and were used as weights to prioritize terms for interpretation.

For each component, the biologically most informative and statistically robust terms were identified by ranking the − log(FDR) scores in descending order and considering their thematic consistency across multiple annotation sources. Terms falling below the 1.3 threshold were noted but de-emphasized in the final interpretation unless they provided important contextual information. We synthesized the top statistically significant and thematically consistent terms into a single descriptive label per inflammatory profile. The label was constructed to reflect the primary biological process, pathway, or molecular function represented, with wording chosen to maintain both precision and interpretability.

In some cases, components shared similar dominant terms, such as inflammatory profiles 1 and 4, which were both enriched for cytokine and interleukin signaling terms. To differentiate these, we examined secondary enrichment patterns and relative − log(FDR) magnitudes.

Within each inflammation profile, a Pearson correlation matrix was generated using raw protein abundance values from all animals within genotype groups. Each inflammatory profile was loading into Cytoscape (Version 3.10.3) to construct a validated network and determine protein-protein interactions, where each protein was represented as a node and the interactions were represented by edges. Correlation matrices were also imported into Cytoscape, and each correlation was mapped onto validated edges between nodes. Network topology was computed within Cytoscape using the NetworkAnalyzer tool, yielding measures including number of nodes and edges, average neighbors, network diameter, radius, characteristic path length, clustering coefficient, density, heterogeneity, centralization, and number of connected components. To integrate genotype group-level effects into the network visualization, nodes were color-coded according to fold-change in protein abundance by genotype (red = increase, blue = decrease), and node sizes were based on loading score. To balance discovery sensitivity with error control in this small-scale protein-protein interaction study, we applied a 10% false discovery rate (FDR) threshold to reduce the likelihood of false negatives and prioritize the identification of biologically meaningful signals for future confirmatory investigation. As such, significant correlations (FDR q < 0.1 adjusted; p_(adj)_) were mapped onto corresponding edges, allowing visualization of protein-behavior relationships within the context of each network. Edge thickness was scaled by the magnitude of the correlation coefficient (thicker lines = stronger *r*); corresponding p_(adj)_-values were mapped as line type (solid = significant, dash = non-significant); the Z-score method^55–57^ was used to calculate observed Z-change in correlation coefficients between WT and TgAD and mapped as edge color (red = more positive change, blue = more negative change); finally, p_(adj)_-values related to observed Z-score changes^58–61^ were scaled as edge transparency (opaque = significant p_(adj)_-vales, transparent = trending or non-significant p_(adj)_-values). Protein-protein association networks for each PCA-derived inflammation profile network were visualized in Cytoscape 3.10.3 using the yFiles Organic layout. This force-directed algorithm treats nodes as mutually repulsive physical objects and edges as metal springs that exert attractive or repulsive forces depending on their length. The iterative simulation arranges nodes such that the overall system reaches a local minimum of total force, producing an undirected, biologically intuitive network layout^62^.

#### Multivariate Analyses of Protein Abundance by Genotype and Sex.

Group differences in inflammatory profile scores were assessed using multivariate ANOVA (MANOVA) with genotype and sex as fixed factors^63^. To evaluate how genotype, sex, and their interaction influenced serum protein levels within each PCA-derived inflammation profile, a MANOVA including all inflammation profile was conducted, with inflammation profile regression scores as dependent variables. Genotype (WT vs. TgAD) and sex (male vs. female) were entered as fixed factors, and the genotype × sex interaction term was included. Multivariate effects were evaluated using Wilks’ Lambda (λ) as the primary test statistic, with Pillai’s Trace, Hotelling’s Trace, and Roy’s Largest Root examined for consistency. When a MANOVA revealed a significant effect of genotype, sex, or their interaction, follow-up univariate ANOVAs were performed to identify the specific proteins driving the multivariate effect. This approach enabled detection of both network-level patterns and the individual protein contributors to genotype- and sex-related differences in inflammatory signaling.

#### Evaluation of associations between inflammation profiles and behavioral performance.

Associations between inflammation profiles and behavioral performance were examined using hierarchical linear regression analyses in IBM SPSS Statistics (Version 29). For each behavioral measure (intertemporal choice, progressive ratio, set-shifting, and delayed match-to-sample), genotype (WT vs. TgAD) and sex (male vs. female) were entered as fixed factors in Model 1 to account for variance attributable to these variables. In Model 2, the regression scores for the relevant PCA-derived inflammation profiles were added as a predictor to assess its unique contribution to behavioral performance after controlling for genotype and sex.

To visualize the unique associations, we extracted isolated residuals for both variables of interest (behavioral performance and inflammation profile scores) representing the variance remaining after statistically accounting for all other predictors in the model. Scatterplots were generated with residuals from the inflammation profile scores on the x-axis and residuals from the behavioral performance measures on the y-axis, thereby illustrating the association between the two variables independent of genotype, sex, and other factors included in the model.

## Supplementary Material

Supplementary Files

This is a list of supplementary fi les associated with this preprint. Click to download.

• SupplementaryTable1Network1KEGGGOTerms.xlsx

• SupplementaryTable2Network2KEGGGOTerms.xlsx

• SupplementaryTable6Allnodes.xlsx

• SupplementaryTable4Network4KEGGGOTerms.xlsx

• SupplementaryTable3Network3KEGGGOTerms.xlsx

• SupplementaryTable8RawData.xlsx

• SupplementaryTable5Network5KEGGGOTerms.xlsx

• SupplementaryTable7AllEdges.xlsx

• SupplementaryTable9GeneProteinNames.xlsx

## Figures and Tables

**Table 1.** Inflammatory Profiles.

Profile	Label	Biological Interpretation
**1**	Systemic pro-inflammatory cytokine receptor signaling	High loadings from proinflammatory cytokines (TNFα, IFNγ, IL-1β, IL-2, IL-4, IL-10, IL-13) and activation markers (CD86, IL-1α, MCP-1, Fractalkine), with growth/neurotrophic factors (Beta-NGF, CNTF, PRLR) and matrix regulators (TIMP-1). Enrichr results showed very high enrichment for broad cytokine receptor signaling processes, indicating systemic interleukin-driven immune activation.
**2**	Cytokine-linked hematopoietic signaling in cancer pathways	Proteins involved in chemotaxis, extracellular matrix regulation, and tissue remodeling (Eotaxin, RANTES, Decorin, FLT3 ligand, Galectin-1, Gas1), plus stromal/progenitor signaling (HGF, Nope, Notchl). Enrichr terms indicated immune-hematopoietic processes and oncogenic signaling, consistent with immune-driven tissue remodeling.
**3**	Anti-inflammatory cytokine signaling in oxidative stress response	Proteins linked to endothelial activation, oxidative stress, and innate immune signaling (IL-6, ICAM-1, PDGF-AA, RAGE, L-selectin, CXCL5, CCL27). Enrichr enrichment for rheumatoid arthritis, cytokine activity, cellular oxidative stress responses, and IL-10 signaling suggests anti-inflammatory modulation within oxidative stress contexts.
**4**	Extracellular matrix-associated interleukin signaling	Proteins associated with epithelial repair, mucosal immunity, and barrier protection (IL-22, IL-17F, IL-7, SCF, TREM-1, P-cadherin). Enrichr indicated interleukin signaling with moderate enrichment values and inclusion of extracellular matrix terms, suggesting cytokine signaling in tissue remodeling and barrier maintenance.
**5**	Metalloproteinase inhibition in immune regulation	Selective proteins (Activin A, Neuropilin-2, Prolactin, TIM-1, CD8O, CINC1) implicated in immune-endocrine signaling, developmental regulation, and tissue repair. Enrichr enrichment for metalloproteinase inhibition and immune activation indicates proteolytic control during immune responses and repair processes.

## Data Availability

All data generated or analyzed during this study are included in this published article.

## Abbreviations

AD: Alzheimer’s disease
TgAD: Transgenic Fisher344-Alzheimer’s Disease rat
